# Supplementary material for: Asymmetric Synthesis of Quaternary Hydantoins via a Palladium-Catalyzed Aza-Heck Cyclization
Source: J Am Chem Soc. 2025 Nov 14;147(49):44692–8. doi: 10.1021/jacs.5c16022 (PMC12703750; doi:10.1021/jacs.5c16022)
Supplement: Supplementary file 2 [file ja5c16022_si_002.zip › All NMR FID Files/S33/S33_ProtonNMR/1/pdata/1/email_EM05-089-1H-DMSO_1_1.pdf]

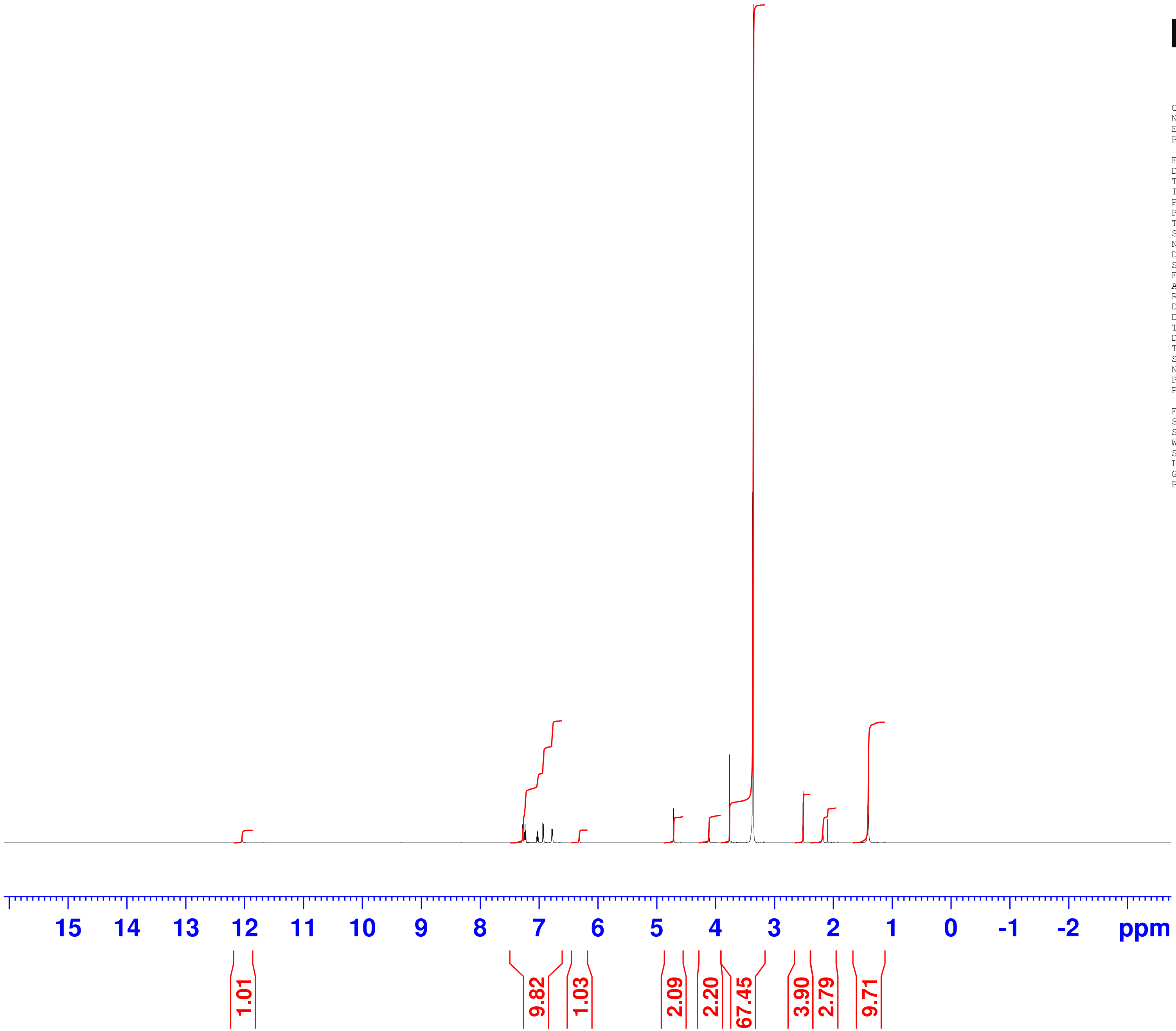

BRUKER

```
Current Data Parameters
NAME       EM05-089-1H-DMSO
EXPNO                     1
PROCNO                    1

F2 - Acquisition Parameters
Date_              20240221
Time                  15.23 h
INSTRUM   Avance Neo 600 at University of Delaware
PROBHD    Z185170_0001 (
PULPROG                  zg
TD                    65536
SOLVENT                DMSO
NS                       16
DS                        2
SWH               11904.762 Hz
FIDRES             0.363304 Hz
AQ                2.7525120 sec
RG                     11.3
DW                   42.000 usec
DE                    10.65 usec
TE                    298.0 K
D1              10.00000000 sec
TD0                       1
SFO1            600.1337058 MHz
NUC1                     1H
P1                     8.01 usec
PLW1             6.90000010 W

F2 - Processing parameters
SI                    65536
SF              600.1300000 MHz
WDW                      EM
SSB                       0
LB                     0.30 Hz
GB                        0
PC                     1.00
```
